# Implementing the Keele stratified care model for patients with low back pain: an observational impact study

**DOI:** 10.1186/s12891-017-1412-9

**Published:** 2017-02-03

**Authors:** Adrian Bamford, Andy Nation, Susie Durrell, Lazaros Andronis, Ellen Rule, Hugh McLeod

**Affiliations:** 1 Gloucestershire Hospitals NHS Foundation Trust, Gloucester, UK; 2 University of Birmingham, Birmingham, UK; 3 Gloucestershire Clinical Commissioning Group, Gloucester, UK

**Keywords:** Low back pain, Stratified care model, STarT Back Screening Tool, IMPaCT Back, Implementation study, Physiotherapy, Economic evaluation

## Abstract

**Background:**

The Keele stratified care model for management of low back pain comprises use of the prognostic STarT Back Screening Tool to allocate patients into one of three risk-defined categories leading to associated risk-specific treatment pathways, such that high-risk patients receive enhanced treatment and more sessions than medium- and low-risk patients. The Keele model is associated with economic benefits and is being widely implemented. The objective was to assess the use of the stratified model following its introduction in an acute hospital physiotherapy department setting in Gloucestershire, England.

**Methods:**

Physiotherapists recorded data on 201 patients treated using the Keele model in two audits in 2013 and 2014. To assess whether implementation of the stratified model was associated with the anticipated range of treatment sessions, regression analysis of the audit data was used to determine whether high- or medium-risk patients received significantly more treatment sessions than low-risk patients. The analysis controlled for patient characteristics, year, physiotherapists’ seniority and physiotherapist. To assess the physiotherapists’ views on the usefulness of the stratified model, audit data on this were analysed using framework methods. To assess the potential economic consequences of introducing the stratified care model in Gloucestershire, published economic evaluation findings on back-related National Health Service (NHS) costs, quality-adjusted life years (QALYs) and societal productivity losses were applied to audit data on the proportion of patients by risk classification and estimates of local incidence.

**Results:**

When the Keele model was implemented, patients received significantly more treatment sessions as the risk-rating increased, in line with the anticipated impact of targeted treatment pathways. Physiotherapists were largely positive about using the model. The potential annual impact of rolling out the model across Gloucestershire is a gain in approximately 30 QALYs, a reduction in productivity losses valued at £1.4 million and almost no change to NHS costs.

**Conclusions:**

The Keele model was implemented and risk-specific treatment pathways successfully used for patients presenting with low back pain. Applying published economic evidence to the Gloucestershire locality suggests that substantial health and productivity outcomes would be associated with rollout of the Keele model while being cost-neutral for the NHS.

## Background

Low back pain (LBP) has reached epidemic proportions, affecting a large number of people each year and with a lifetime prevalence of up to 80% [1, 2]. Each year in England, about 2.3 million people, 4.2% of the population, consult a general practitioner (GP) at least once for LBP [3], and these individuals represent about 20% of those with LBP [4]. Back pain causes considerable discomfort and is a leading cause of work absence and economic loss [1, 5]. UK costs associated with back pain were conservatively estimated in 1998 prices as follows: NHS treatment £1.1 billion, private treatment £0.6 billion, informal care £1.6 billion, employment-related productivity loss costs £3.4 billion [1]. These figures underline the impact of LBP on society.

A wide range of therapies have been used for treatment of LBP [6], although clinicians’ views on appropriate therapies have varied [7] along with reported uncertainty about the value of treatment options [8] and calls for research on cost-effectiveness [9]. The type of treatment for which there is most economic evidence is combined physical and psychological interventions. These include those that are physiotherapist-led (spanning the use of the Keele stratified care model [10–13], group exercise and education sessions [14], two pain-management programmes [15, 16] and spinal stabilisation physiotherapy [16]), multidisciplinary (with input from different combinations of physiotherapists, nurses, psychologists or professional counsellors) [17–20], and a psychologist-led intervention [21]. With one exception [15], the interventions have been compared to usual care and found to be cost-effective. However, the main evaluation perspectives taken in these studies vary from healthcare system (such as the NHS) to healthcare (system and private) to societal (healthcare and productivity losses), and this makes it difficult for clinicians and local commissioners to compare treatment options.

### The Keele stratified care model

The Keele stratified care model for management of LBP comprises use of the prognostic STarT Back Screening Tool to allocate patients into one of three risk-defined categories [22] followed by delivery of associated risk-specific treatment pathways [10]. All patients receive a 30-min session with a physiotherapist comprising reassurance, education, and treatment specific to the patient’s screening tool score [23]. Low-risk patients receive no further intervention. Medium-risk patients present with predominantly physical prognostic indicators and are referred for further physiotherapy. The high-risk patients are also referred for further physiotherapy which is designed to address the high psychosocial indicators, such as anxiety and fear, with which they present. A training package is required for the physiotherapists to enable them to deliver the treatment pathways [23]. The intention of the stratified care model was to change the pattern of treatment such that it would better target appropriate interventions and improve patient outcomes. The initial randomised controlled trial (the STarT Back trial) comparing the stratified care model to usual care was designed so that all eligible LBP patients identified from routine general practice records were seen in a physiotherapy clinic [10]. This does not reflect usual practice, where GPs are the first-contact clinician and only a minority of LBP patients are referred to physiotherapy [10]. The subsequent IMPaCT Back study was a sequential comparison study designed to explore the impact of the Keele stratified care model when implemented by GPs [12, 13]. Both the STarT Back and IMPaCT Back studies found that the stratified care model’s treatment pathways change the pattern of usual treatment resulting in an increase in the number of treatment sessions provided as the risk-classification increases. The IMPaCT Back study found that over a 6-month post-implementation period (phase 3) the stratified care model was associated with a gain in mean quality-adjusted life years (QALYs) of 0.003 per patient compared to a 6-month pre-implementation period (phase 1) [12]. An increase in mean back-related NHS costs over 6 months of £1.75 per patient was associated with stratified care compared to a reduction in mean costs of £33.54 when private treatment costs were included [12, 13]. Stratified care was also associated with a mean saving in indirect productivity loss costs associated with LBP-related work absence of £400 per employed patient over the 6-month period [12].

### Aims

The Keele stratified care model has attracted considerable NHS [24] and international [25] interest since the initial randomised controlled trial findings were published in 2011 [10], but there is little published information on its performance and usefulness in everyday practice [24, 25]. With this in mind, we set out to assess the impact of the model following its implementation as a pilot by physiotherapists at Gloucestershire Hospitals NHS Foundation Trust (GHT), an acute hospital physiotherapy department setting in Gloucestershire, England, in 2013. Use of the stratified care model is assessed in terms of three criteria: evidence of i) whether it was associated with the anticipated treatment pathways as represented by the number of risk-specific treatment sessions provided, ii) the physiotherapists’ views on the usefulness of the stratified care model because the model is more likely to inform clinical behaviour if it is viewed as useful by the physiotherapists, and iii) the potential consequences for NHS and private treatment costs, QALYs, and societal productivity losses associated with the rollout of the stratified care model in Gloucestershire.

## Methods

### Design and setting

Treatment for LBP in Gloucestershire is provided by physiotherapists in the outpatient departments of both the local acute hospital and community care provider Trusts. Referrals to both services are received either from a GP or patient self-referral. The acute hospital physiotherapy service introduced the Keele stratified care model in Spring 2013, when patients presenting with LBP at one of the two acute hospital sites started to complete the prognostic STarT Back Screening Tool to determine their risk-rating at the first physiotherapy appointment. In order to audit this service innovation, an audit was undertaken during 15 weeks from March 2013 and repeated during 8 weeks from June 2014, by AN as part of the Trust’s empowered leadership programme. The audit data were essential for the study because diagnostic codes are not routinely recorded on electronic systems for outpatient attendances at the Trust and so in their absence it would have been necessary to examine paper patient physiotherapy records to identify patients.

The GHT audit data allow an observation cohort design for the study, which also draws on the findings of the IMPaCT Back sequential comparison study to assess two of the study’s aims: whether the introduction of the stratified care model was associated with the anticipated treatment pathways, and what the potential consequences of rolling out the model in Gloucestershire would be for NHS and private treatment costs, QALYs, and societal productivity losses.

### Data

In each audit, physiotherapists were requested to complete an audit form on 100 consecutive patients for whom the patients’ risk-rating had been determined. The sample size was determined on pragmatic grounds to be logistically feasible. The data recorded included: patient hospital number, Keele STarT Back Screening Tool score (1 to 9), risk-defined category (low, medium, high), clinical impression/differential diagnosis, number of treatment sessions, type of treatment, outcome, ‘yes’ or ‘no’ response to the question ‘In your opinion did the risk category accurately reflect the patient?’, free text response to ‘Reflection - usefulness of the tool/did it guide patient management etc’. The audit forms were logged on a summary sheet that included a physiotherapist identifier code and the date on which the patient was first seen by a physiotherapist. A classification code for the physiotherapists’ seniority was added to the dataset on the basis of the recorded physiotherapist identifier code by the head of service (SD), to distinguish physiotherapists up to grade 5 from those in higher grades. Where possible, the patient hospital number and the initial attendance date were used to determine the patient gender and age in years from the electronic patient administration system. The 2013 audit data for a small number of patients were recorded before the patient was discharged and so, where possible, the patient hospital number and the initial attendance date were used to determine the number of physiotherapy sessions undertaken from the patient administration system.

### Analysis

Statistical analyses was used to assess whether implementation of the stratified care model was associated with the anticipated treatment pathways as represented by the number of risk-specific treatment sessions provided. This involved i) generating descriptive statistics for the number of treatment sessions provided and comparing them to those for the STarT Back and IMPaCT Back studies, ii) carrying out regression analysis using a multilevel linear mixed-effects model to examine the relationship between the patient-level number of treatment sessions and the risk-defined category, controlling for patient age, gender, year of audit, physiotherapists’ seniority and physiotherapist. The risk-defined category was included as a fixed-effect categorical variable, and the patient age in years was included as a fixed-effect continuous variable. Gender (1 = female, 2 = male), year of audit (1 = 2013, 2 = 2014), and physiotherapists’ seniority (1 = up to grade 5, 2 = higher grades) were included as fixed-effect dichotomous variables. Physiotherapist was included as a random-effect categorical variable in order to account for a possible physiotherapist-level cluster effect. Log transformation of the treatment session data was undertaken in order to reduce the right-skewness of the distribution due to the small number of patients receiving a comparatively large number of treatment sessions. The Kernel density estimate of the treatment session square root transformation was closer to normality compared to the Kernel of either the square root transformation or the natural units. The exponential of each fixed-effect coefficient is reported, as it represents the expected percent change in the number of treatment sessions associated with a one unit change in the independent variable [26]. Statistical significance was set at the 5% level. STATA version 12 software was used for the analysis. In order to provide an alternative measure of physiotherapist-level effects, a linear fixed-effects regression model was also run. However, the results were not sensitive to choice of model and so the results of the linear fixed-effects regression model are not reported here. Sensitivity analysis included omitting physiotherapist as an independent variable. A small-scale data validation exercise was undertaken for six patients to check the extent to which the physiotherapist’s record of the number of physiotherapy sessions matched the medical records and number of sessions recorded in the electronic patient administration system. No evidence of discrepancies was found.

Audit data on whether the physiotherapist thought the risk category accurately reflected the patient were analysed to assess the percentage of patients and mean number of treatments by risk group to see if there was a link between valuing the tool and adopting a treatment strategy that reflected the risk category it produced. To further explore the physiotherapists’ views on the usefulness of the stratified care model, the free text data on this question were analysed using framework methods [27]. Framework uses a thematic structure to summarise and classify case-based data that are organised in a series of matrices. Text is coded to represent key topics/themes and recorded in columns, with rows representing individual informants. This facilitates exploration of themes and patterns across the range of informants. In this study, the physiotherapists’ views were first classified by whether the physiotherapist viewed the risk-rating tool as accurately reflecting the patient. The physiotherapists’ comments were then coded as ‘useful’ or ‘not useful’ before associated attributes were identified.

The potential consequences for NHS and private back-related treatment costs, QALYs, and societal productivity losses associated with the rollout of the stratified care model in Gloucestershire were modelled by applying the local audit data on the proportion of patients by risk classification and estimates of local incidence to the published 6-month parameter estimates from the IMPaCT Back study [13]. Costs included consultations with GPs and practice nurses, consultations with other healthcare professionals, hospital-based procedures, prescribed medication, and out-of-pocket expenditures on treatments and/or aids [13]. The impact on treatment costs is reported from the perspectives of the NHS, private, and healthcare (NHS and private). Productivity losses are reported from a societal perspective. The impact of the stratified care model can be expressed in terms of net monetary benefit (NMB). The incremental benefit is rescaled into monetary value using the cost-effectiveness threshold to value each unit of benefit, and the incremental cost is subtracted from this value to give the NMB [28]. The cost-effective threshold used by the National Institute of Health and Care Excellence (NICE) is £30,000 per QALY, and this value is used in our analysis [28]. As a sensitivity analysis we also apply a cost-effective threshold of £15,500 per QALY which was empirically estimated for musculoskeletal treatment by Claxton et al. [29] and reported by Drummond et al. [28] table 4.3.

The proportions of patients by risk classification were estimated from the GHT audit data. The annual number of LBP patients referred from general practice for physiotherapy treatment was estimated as 20% [12, 30] of the consulting prevalence for LBP from an observational study of primary care consultations [3], applied to Gloucestershire gender and age-specific population rates from Office for National Statistics mid-year estimates for 2013 [31]. The estimate of 20% of the consulting prevalence was increased to 25% in a sensitivity analysis. The 2008/9 back-related costs from the IMPaCT Back study [13] were translated into 2013/14 values using the hospital and community health services (HCHS) index to account for pay and price inflation [32]. A sensitivity analysis of the percentage of patients in each risk-category was undertaken assuming the percentages from the IMPaCT Back study in place of the GHT audit findings.

## Results

The GHT audits included 201 patients presenting with LBP, of whom 59% (118/201) were female. Patient age was recorded for 92% (184/201) of the patients and the mean age was 51 years (range 16 to 90 years). The 201 patients were seen by 29 physiotherapists; 23 participated in the 2013 audit, 18 in 2014, and 12 in both audits. Patients were randomly allocated to a physiotherapist and on average each participating physiotherapist saw 6.9 patients (range 1 to 36 patients).

### Treatment pathways

After the introduction of the Keele model, 2.7 physiotherapy sessions were provided to the LBP patients, on average, and the trend was for more sessions to be provided on average as the risk-rating increased (Table 1). Similarly, the trend was that more sessions were provided on average in 2014 compared to 2013.

**Table 1:** GHT audits: the number of LBP patients and NHS physiotherapy treatment sessions by risk category and year

stratified care model risk category	2013				2014				total			
	number (%) of patients		mean number (sd) of treatment sessions		number (%) of patients		mean number (sd) of treatment sessions		number (%) of patients		mean number (sd) of treatment sessions	
Low	30	(30)	1.63	(1.2)	29	(29)	2.31	(1.9)	59	(29)	1.97	(1.6)
Medium	36	(36)	2.31	(1.4)	40	(40)	3.18	(3.2)	76	(38)	2.76	(2.5)
High	35	(35)	3.09	(3.0)	31	(31)	3.42	(2.5)	66	(33)	3.24	(2.8)
Total	101	(100)	2.38	(2.1)	100	(100)	3.00	(2.7)	201	(100)	2.69	(2.4)

*sd* standard deviation

The experience of the GHT audit patients was similar to that of patients in the IMPaCT Back study, such that the trend was for more sessions to be provided on average as the risk-rating increased (Table 2). This comparison is based on the 57% (314/554) of patients in the IMPaCT Back phase 3 for whom data on resource use was available (Table 2) [13]. Table 2 also illustrates the impact of the introduction of the stratified care model in the IMPaCT Back study, in terms of the increase in the mean number of treatment sessions, particularly for patients in the medium- and high-risk categories.

**Table 2:** IMPaCT Back study: the number of LBP patients and NHS physiotherapy treatment sessions by risk category

stratified care model risk category	pre-implementation: phase 1						post-implementation: phase 3						% change in mean number of treatment sessions between phases 1 and 3
	all patients^a^		patients providing data at 6 months^b^				all patients^a^		patients providing data at 6 months^b^				
	number (%)		number (%)		mean number (sd) of treatment sessions		number (%)		number (%)		mean number (sd) of treatment sessions		
Low	136	(37)	81	(35)	1.05	(2.3)	214	(39)	110	(35)	1.14	(3.3)	9
Medium	151	(41)	104	(45)	1.45	(0.8)	232	(42)	143	(46)	2.08	(3.0)	43
High	81	(22)	48	(21)	1.87	(2.8)	108	(20)	61	(19)	2.67	(3.9)	43
Total	368	(100)	233	(100)	1.40		554	(100)	314	(100)	1.87		33

*sd* standard deviation

^a^Source: risk-category level data from Hill et al., [10, Table 1]

^b^Source: risk-category level data from Whitehurst et al., [13, web appendix table 3b]

Overall, 33% (66/201) of the patients in the GHT audits were classified as high-risk, compared to 38% medium-risk and 29% low-risk (Table 1). The GHT experience was for a larger proportion of patients to be classified as high-risk compared to the 20% (108/554) experienced in the post-implementation phase of the IMPaCT Back study (Table 2). The chi-squared test indicates that the proportions in each risk category in the GHT audits compared to the post-implementation phase of the IMPaCT Back study were significantly different (*p* < 0.01) (Tables 1 and 2).

The multilevel linear mixed-effects model was used to examine the relationship between the number of treatment sessions and the risk-defined category, while controlling for patient and physiotherapist-level variables (Table 3). The audit year, patient gender and physiotherapists’ seniority were not significant explanatory variables (Table 3). However, compared to patients in the low-risk category, patients in the medium- and high-risk categories received on average 29.3% and 54.6% more treatment sessions, respectively, and these differences were statistically significant (Table 3). This finding confirms that GHT experienced treatment patterns, in terms of the number of physiotherapy sessions provided, in line with the expectations of the stratified care model.

**Table 3:** GHT audits multilevel linear regression model coefficients for log of the number of treatment sessions

fixed effects	coefficient	exponential of coefficient	p value	95% CIs
audit year	0.146	1.157	0.160	−0.058 to 0.350
patient gender	0.061	1.063	0.529	−0.130 to 0.253
physio seniority	−0.132	0.876	0.351	−0.410 to 0.145
Risk (low)				
medium	0.257	1.293	0.032	0.022 to 0.492
high	0.435	1.546	<0.001	0.192 to 0.679
intercept	0.389	1.475	0.184	−0.184 to 0.961
Random effects	estimate	95% CIs		
physiotherapist				
sd cons	0.191	0.077 to 0.470		
sd residual	0.667	0.601 to 0.741		

Running the model with patient age for the 184 patients for whom the data were available did not change the results. In order to provide an alternative measure of physiotherapist-level effects, a linear fixed-effects regression model was also run. The risk-category results were not sensitive to choice of model (patients in the medium- and high-risk categories received on average 31.0% or 61.9% more treatment sessions, respectively, compared to patients in the low-risk category, and these differences were significant at the 5% level), although the audit year variable was also significant and indicated that patients in the 2014 audit received 24.7% more treatment sessions than those in the 2013 audit. Omitting physiotherapist as an independent variable altogether in a fixed-effects model found patients in the high-risk category receiving on average 55.3% more treatment sessions, compared to patients in the low-risk category, and the difference was significant (*p* = 0.01), but the 25.8% increase for medium-risk patients was not significant (*p* = 0.06).

### Physiotherapists’ views on the usefulness of the stratified care model

The audits requested the physiotherapist to record their view on whether or not the risk category accurately reflected the patient, in other words whether they thought that the prognostic risk-rating had aided them in managing the condition, and any comment on the usefulness of the screening tool. These data for the high-risk patients in the 2013 audit were not available for the study and so the analysis of these data is limited to the 2014 audit. In 67% (67/100) of cases the physiotherapist indicated that the screening tool had accurately reflected the patient, compared to 15% taking the opposite view, and 18% not responding to the question (Table 4). When the physiotherapist noted that the screening tool had accurately reflected the patient, the pattern of mean treatment sessions was in line with the expectations of the stratified care model: more sessions were provided to higher-risk patients (Table 4). However, this was not the case when physiotherapist noted that the screening tool had not accurately reflected the patient, or had not responded to the question (Table 4).

**Table 4:** GHT 2014 audit: physiotherapists’ view on whether the risk category accurately reflected the patient by risk category and number of patients and NHS treatment sessions

stratified care model risk category	‘yes’				‘no’				‘no response’			
	number (%) of patients		mean number (sd) of treatment sessions		number (%) of patients		mean number (sd) of treatment sessions		number (%) of patients		mean number (sd) of treatment sessions	
Low	19	(28)	1.6	(1.1)	4	(27)	4.5	(2.4)	6	(33)	3.2	(2.2)
Medium	26	(39)	2.4	(2.3)	6	(40)	2.0	(1.5)	8	(44)	6.6	(4.4)
High	22	(33)	3.5	(2.7)	5	(33)	3.0	(2.4)	4	(22)	3.3	(2.2)
Total	67	(100)	2.5	(2.3)	15	(100)	3.0	(2.2)	18	(100)	4.7	(3.6)

*sd* standard deviation

In the 2014 audit, comments were recorded by 13 of the 18 physiotherapists on their views of the usefulness of the screening tool for 43 of the 100 patients, and other comments were reported for a further 12 cases. Our analysis of the comments focuses first on those cases where the screening tool was viewed as having accurately reflected the patient, before considering those cases where the screening tool was not viewed as having accurately reflected the patient. In the two-thirds of cases when the physiotherapist had indicated that the screening tool had accurately reflected the patient, the comments on usefulness were positive in 93% (28/30) cases. The positive comments applied across risk categories, patient ages, physiotherapist seniority, diagnoses and treatments, and they suggest benefits for both the patient, for example “*prompted discussion about concerns* – *enabling reassurance*”, and physiotherapist, for example “*v useful*, *guided me to give advice & d*/*c* [*discharge*]”. For low-risk patients the feedback suggests that use of the tool guided management and discharge, while for medium- and high-risk patients it facilitated communication between physiotherapists and patients which led to anxiety being identified and then addressed. Two comments were not positive; one was for a low-risk patient presenting with posterior/lateral derangement who received five treatment sessions before being discharged and the physiotherapist’s comment was “*Due to symptoms & progressions of HEP* [*home exercise programme*] *needed low category did not reflect no. of treatments needed. Also had hip issue which needed clarification*”. The other case was a medium-risk patient presenting with mechanical LBP who received one treatment session before being discharged and the physiotherapist’s comment was “*No it didn*’*t guide management*”.

Comments on usefulness were made for 13 of the 15 cases where the physiotherapist had indicated that the screening tool had not accurately reflected the patient. In the four low-risk cases, the physiotherapist viewed the patient as being more anxious or requiring more clinical support than that indicated by the low-risk classification, and in each case the patient received more than one treatment session. Of the six medium-risk cases, comments on two indicated that the risk-rating may not be optimal; being either too high “*More easily reassured than tool score suggested*” or too low “*I feel high risk category may have been more appropriate*”. In two cases, the comments indicated that the use of any care model was not relevant; “*Did not guide treatment as pt v specific about what she wanted*” and “*Not useful as pt seeking advice about falls protection*”. In the other two cases, the comments do not provide insight into why the screening tool was “*not helpful*” or “*not relevant for this pt*”. Of the high-risk patients, two cases suggest that initial patient anxiety was addressed such that the high-risk classification may not have been warranted: “*Anxious due to acute onset but able to reassure*” was the comment for one of patients, who did not attend a second treatment session. The third high-risk case appeared to illustrate inappropriate completion of the screening tool: “*Main problem OA hip & pt misinterpreted tool by answering re hip pain*”. Overall, where the physiotherapist had indicated that the screening tool had not accurately reflected the patient, their comments suggest the risk-rating was viewed as too low in five cases and too high in three cases.

### Potential economic consequences associated with the rollout in Gloucestershire

The IMPaCT Back study estimated the incremental change in NHS costs and healthcare costs (NHS and private) associated with the introduction of the stratified care model using data imputation methods for its full patient sample (*n* = 922) (Table 5) [12]. The changes in these costs by risk category up-rated to 2014/15 values are summarised in Table 5, which shows that applying the percentage of patients in each risk-category experienced in the GHT audits (Table 1), generates an estimated aggregate incremental back-related NHS cost saving of £0.65 per patient associated with the stratified care model. Similarly, applying the IMPaCT Back study’s estimated incremental change in QALYs to the percentages of patients in each risk-category experienced in the GHT audits gives an estimated aggregate incremental QALY gain of 0.006 per patient associated with the stratified care model (Table 5). The estimated annual number of patients referred for LBP physiotherapy treatment in Gloucestershire is 5217 patients (Table 5), which suggests a NHS cost saving of approximately £3400 per annum and an associated gain of approximately 30.2 QALYs and a net monetary benefit of approximately £909,000. The NMB is based on the cost-effectiveness threshold associated with NICE of £30,000 per QALY, and an alternative empirically estimated threshold of £15,500 per QALY for musculoskeletal treatments implies associated NMB of £471,000.

**Table 5:** Estimated change to back-related NHS costs and QALYs associated with introducing stratified care in Gloucestershire

stratified care model risk category	% patients in risk category^a^	mean incremental costs and QALYs per patient over 6 months associated with the stratified care model:^b^				annual referrals for LBP physio. in Glos.^c^	annual incremental impact for Gloucestershire:		
		Costs (£)			QALYs		NHS cost (£ 1000)	QALY gain	NMB^d^ (£ 1000)
		NHS	private	total					
Low	29	−6.58	9.85	3.27	0.003	1531	−10.1	4.6	147.9
Medium	38	14.96	−23.52	−8.56	−0.007	1973	29.5	−13.8	−443.8
High	33	−13.33	−122.61	−135.93	0.023	1713	−22.8	39.4	1204.8
Total	100	−0.65	−46.26	−46.91	0.006	5217	−3.4	30.2	909.0

*physio* physiotherapy, *Glos* Gloucestershire, *NMB* net monetary benefit

^a^Source: GHT audits (Table 1)

^b^Source: IMPaCT Back study [13, Table 3]. Costs up-rated to 2014/15 values using the HCHS index [31]: private costs extrapolated from NHS and total (healthcare) costs

^c^Source: 20% [10, 30] of 26,085, the estimated annual consulting prevalence for LBP in primary care [3] applied to Gloucestershire gender and age-specific population rates for 2013 [31]

^d^assuming a cost-effectiveness threshold of £30,000 per QALY

The estimate of the annual number of patients referred for LBP physiotherapy treatment may be conservative; it assumes that 20% of the estimated annual consulting prevalence for LBP in primary care is referred. In practice, for example, some LBP patients may be referred for physiotherapy more than once in a year. If 25% of the estimated annual consulting prevalence for LBP in primary care is referred, the annual NHS cost saving would be approximately £4200 with an associated gain of 37.7 QALYs and NMB of £1,136,000 (or £589,000 based on the alternative empirically estimated cost-effectiveness threshold).

These findings are sensitive to the percentage of patients in each risk-category; applying those for all patients in the post-implementation phase of IMPaCT Back study (Table 2) result in an overall increase in mean back-related NHS costs of £1.13 per patient (£1.03 up-rated to 2014/15 values). This indicates an increase in NHS costs of approximately £5900 and an increase in 14.1 QALYs and NMB of £418,000 (or £213,000 based on the empirically estimated threshold) in Gloucestershire.

The IMPaCT Back study reported productivity losses associated with LBP-related work absence following the introduction of the stratified care model [13], which suggests a mean saving of £545 per employed patient (in 2014 values) (Table 6). Applying the IMPaCT study’s estimates of the percentage of LBP patients in work, and the mean change in the number of days of LBP-related work absence during the 6 months following the introduction of the stratified care model, to the estimated annual number of patients referred for LBP physiotherapy treatment in Gloucestershire, suggests a saving of approximately 10,500 days of work absence valued at £1.4 million (Table 6).

**Table 6:** Estimated change to productivity costs associated with introducing stratified care in Gloucestershire

Risk category	IMPaCT Back study estimates^a^			Gloucestershire estimates		
	% of LBP patients in work	change in mean no days off work for employed patients	Change in mean cost of LBP-related work absence^b^ (£)	Number of LBP patients in work^c^	mean change in no of days of LBP-related work absence	Change in cost of LBP-related work absence (£ million)
Low	57	0.3	94	873	288	0.1
Medium	48	−6.1	−909	947	−5785	−0.9
High	48	−6.1	−805	822	−4975	−0.7
Total	51	−4.0	−545	2642	−10,472	−1.4

^a^Source: [13, Table 4]

^b^Costs over 6 months up-rated to 2014 prices using the retail price index [32]

^c^Applying the IMPaCT Back study’s estimate of the percentage of LBP patients in work and the annual estimated annual referrals for LBP physiotherapy in Gloucestershire (Table 5)

As above, assuming a higher estimate of incidence of 25% of the annual consulting prevalence indicates a productivity loss saving of approximately 13,100 days of work absence valued at £1.8 million. Alternatively applying the percentage of patients in each risk-category from the IMPaCT Back study post-implementation phase (Table 2) result in a mean saving of £461 per employed patient (in 2014 values), and an aggregate productivity loss saving of approximately 9000 days of work absence valued at £1.2 million.

## Discussion

In 2013, the physiotherapy department at GHT started to use the stratified care model for LBP patients, with the expectation that prognostic screening and matched treatment pathways would improve the pattern of activity, such that at the extremes low-risk patients would be appropriately discharged after a single treatment session, and high-risk patients would receive a psychologically-informed treatment package to address psychosocial obstacles to recovery as well as physical symptoms and function [10]. The analysis of the GHT audit data indicates that the use of the stratified care model was broadly associated with the anticipated pattern of treatment sessions. On the whole, patients received more treatment sessions as the patients’ risk-rating increased, having controlled for patient and physiotherapist characteristics, which suggests that the stratified care model was supporting the intended treatment practice.

The physiotherapists’ audit feedback on the model confirmed that the risk-rating was viewed as accurately reflecting the patient in 82% (67/82) of the cases where data were reported. Comments on the cases where the patient was not viewed as being accurately reflected by the risk-rating indicated a few cases where the physiotherapist felt that a different rating would have been appropriate. One of the factors cited relates to patient anxiety, and to the extent that its impact can be addressed, the findings suggest that it is important for physiotherapists to have appropriate specialist training. When the physiotherapists viewed the prognostic risk-rating as having aided them in managing the LBP, the mean number of treatment sessions was 1.6, which indicates that many of these low-risk patients were discharged after one session in line with expectations of the Keele model. The audit data also show that there were a few instances where the model was not viewed as relevant because, for one reason or another, the patient was not primarily seeking treatment for LBP. This issue may have influenced the omission of recording a response to the audit question on whether or not the tool accurately reflected the patient. If we assume that all non-responses to this question indicated that the risk-rating was not viewed as accurately reflecting the patient, then in 67% (67/100) of the cases the audit feedback on the model confirmed that the risk-rating was viewed as accurately reflecting the patient. When the physiotherapists’ gave feedback on the prognostic screening tool it was dominated by positive views, which represent an important component of this impact assessment.

The potential economic consequences associated with the introduction of the stratified model in Gloucestershire were estimated by applying published economic evaluation findings on back-related NHS costs, QALYs and societal productivity losses to the local experience of the proportion of patients in each risk category, and estimates of local incidence. This approach suggests that the introduction of the model could achieve an annual incremental gain of about 30 QALYs generating a net monetary benefit of £471,000, on the basis of an empirically-estimated cost-effectiveness threshold of half the level indicated by NICE. Looking beyond benefits associated with health-related quality of life, the economic evaluation evidence suggests that a societal benefit from fewer work days lost because of LBP corresponds to a productivity loss saving of £1.4 million which could be achieved by rolling out the model in Gloucestershire. The economic evaluation evidence suggests that these benefits are associated with almost no impact on NHS costs.

The strengths of the study lie in its analysis combining local audit data and published economic evaluation results to assess the potential of the stratified care model in a physiotherapy department setting. The analysis of the physiotherapists’ views of the risk-rating is a strength of the study, albeit limited by the necessity to focus on the 2014 audit data. The analysis demonstrates a pragmatic approach to applying trial findings to a real world scenario. The analysis is limited by its reliance on the audits to collect data on use of the stratified care model, and literature-based estimates of the number of LBP patients receiving physiotherapy. While our findings may not be generalisable to other settings, our analytical approach could be widely employed to assess the potential impact of the stratified care model.

The extent to which the IMPaCT Back study can inform the potential impact of introducing the stratified care model in a setting such as Gloucestershire depends on the extent to which the study’s experience is likely to be relevant. The general context is similar in terms of both settings being in the NHS in England, and the IMPaCT Back study being based on largely urban general practices in Staffordshire, while the Gloucestershire audits were based on patients mostly from the Gloucester locality. However, the IMPaCT Back study involved GPs administering the screening tool and hence their decisions to refer patients for physiotherapy, whereas in Gloucestershire the tool was administered by physiotherapists following GP referral, or in some cases, patient self-referral. This means that the denominator in each setting is different; the GHT patients represent a subset of comparatively higher-risk referred patients, compared to those in the IMPaCT Back study. This difference in context may account for the 39% low-risk patients in the phase 3 IMPaCT Back study compared to 29% in the GHT audits. The consequences are appreciable; the GHT risk-specific percentages are associated with an additional net monetary benefit of £201,000 compared to the phase 3 IMPaCT Back study, based on the conservative empirically-estimated cost-effectiveness threshold. To the extent that the stratified care model may for logistical reasons be more likely to be implemented by physiotherapists for referred patients, rather than by GPs in primary care, the GHT experience provides new insight into the scale of the stratified care model’s potential benefits. Having noted the similarities and differences in context between the IMPaCT Back study and the use of the tool in Gloucestershire, we view that the risk-level treatment effects found in the IMPaCT Back study provide a reasonable, and certainly the best available, basis for estimating the potential consequences for rolling out the stratified care model in Gloucestershire. The IMPaCT Back study found that pre-implementation of the Keele model, the mean number of treatment sessions increased with the risk rating, and that after the introduction of the Keele model, patients with higher risk-ratings received comparatively more treatment sessions. We assume that GHT will experience similar risk-level treatment effects. Better estimates of impact would require a local cost-utility analysis, entailing collection of data on resource use, costs and health-related quality of life, both before and after the introduction of the Keele model and for a suitable control group.

Being a sequential comparison study, rather than a randomised trial, the IMPaCT Back study must be considered with caution. Nevertheless, its findings about the risk-specific incremental impact on NHS costs and outcomes offer a guide for likely experience in Gloucestershire. From a commissioner’s decision-making perspective, the IMPaCT Back study’s main analysis would have been even more informative if it had been undertaken from an NHS perspective, in line with NICE guidance [33], rather than a healthcare perspective. The reported healthcare cost savings are due to a reduction in mean private treatment costs largely experienced by high-risk patients (Table 5). The inclusion of private costs in the main Keele economic evaluations mean that the analysis of uncertainty, using cost-utility planes and cost-effectiveness acceptability curves, may be of comparatively limited value for commissioners. Furthermore, while the IMPaCT Back study’s reporting at risk-defined subgroup level emphasises the dominance of the intervention for high-risk patients, and the opposite for medium-risk patients, our analysis indicates that the stratified care model is dominant for the whole patient group represented by the GHT audits (i.e. reduced NHS costs, albeit very slightly, and gains in health-related quality of life). Economic theory indicates that the uncertainty relating to this outcome, where differences in cost and QALYs are not statistically significant [13] should not impede implementation, but should prompt consideration of commissioning a value of information study to assess whether further research should be undertaken [34]. In this instance, a pragmatic response would be to focus future local quality improvement effort on ensuring that the treatment package for medium-risk patients has an optimal impact on health-related quality of life. This would be facilitated by routine coding of outpatient attendances by clinical diagnosis and, when used, the risk-stratification scores.

The estimated net monetary benefit associated with the adoption of the stratified care model is substantial, despite the estimated mean incremental QALY gain being modest over 6 months (0.006), because of the large number of patients and small cost consequences (Table 5). This raises the question of whether other LBP interventions are associated with larger impact on health-related quality of life. The NHS advice, assessment and group cognitive behavioural therapy (CBT) intervention reported by Lamb et al. [19, 35, 36] stands out for achieving a comparatively large incremental QALY gain of 0.099 over 12 months. However, comparison with the stratified care model is not straightforward. First, a follow-up study of 56% (395/701) of the original trial participants, who were older and had less disability and pain at baseline compared to the full patient cohort, found no incremental gain in EQ5D scores at 3, 6, 12 or the average follow-up period of 34 months [36]. This suggests that as for the stratified care model, QALY gains may be mostly attributable to those patients with a poorer prognosis. Second, the CBT study reported a secondary analysis of costs, aiming to reflect the likely costs for NHS rather than those associated with the delivery of the trial, which estimated a £96 incremental NHS cost over 12 months largely due to the £126 intervention cost per patient [35]. These costs represent a considerable additional resource requirement in comparison to the stratified care model of about £497,000 in Gloucestershire. Commissioners may favour the stratified care model in this case because of the NHS resource consequences. However, the CBT intervention’s estimated costs may not be readily comparable with those of other non-group-based interventions. This is because it allocated session costs to the number of patients that attended, so the cost of resource allocated to the patients who did not attend sessions were included. In contrast, the cost of resource allocated to the patients who did not attend booked physiotherapy sessions in the stratified care model studies were not included. Furthermore, it may be that the CBT intervention could be targeted and delivered in fewer sessions, as only 62% of participants attended at least 3 of the 6 group sessions held [35]. Overall, it would be interesting to see whether the stratified care model’s impact on health-related quality of life could be enhanced by combining the risk-stratification tool with targeted use of group CBT. The draft guideline on the management of non-specific LBP published by the National Clinical Guideline Centre in February 2016 endorsed consideration of the STarT Back risk assessment tool for risk stratification along with a range of potential treatment options including psychological therapies for managing LBP as part of multi-modal treatment packages or combined physical and psychological programme for people with persistent LBP [37].

## Conclusion

The Keele stratified care model was implemented and risk-specific treatment pathways successfully used for patients presenting with LBP for physiotherapy in Gloucestershire. The physiotherapists’ feedback on using the prognostic screening tool was very positive. Applying published economic evidence to the Gloucestershire locality suggests that substantial health and productivity outcomes would be associated with rollout of the Keele model while being largely cost-neutral for the NHS.

## Abbreviations

CBT: Cognitive behavioural therapy
GHT: Gloucestershire Hospitals NHS Foundation Trust
GP: General practitioner
HCHS: Hospital and community health services
HEP: Home exercise programme
LBP: Low back pain
NICE: National Institute of Health and Care Excellence
NMB: Net monetary benefit
QALYs: Quality-adjusted life years
